# Bright paint makes interior-space surfaces appear farther away

**DOI:** 10.1371/journal.pone.0201976

**Published:** 2018-09-05

**Authors:** Christoph von Castell, Heiko Hecht, Daniel Oberfeld

**Affiliations:** Johannes Gutenberg-Universität Mainz, Mainz, Germany; Birkbeck University of London, UNITED KINGDOM

## Abstract

Previous studies have reported that bright ceilings appear higher than dark ceilings, irrespective of the other colorimetric properties of the ceiling color (hue, saturation) and irrespective of the luminance of the remaining room surfaces (walls, floor). In the present study, we expand these findings to width and depth estimates. We presented stereoscopic full-scale room simulations on a head-mounted display and varied the luminance of the side walls, rear wall, and ceiling independently of each other. Participants judged the width and depth of the simulated rooms. Our results show that the perceived spatial layout of a given room is significantly influenced by the luminance of the direct bounding surfaces (e.g., the side walls when judging perceived width) but less affected by the luminance of the other surfaces. In the discussion, we provide an overall picture of effects of surface luminance on the perceived layout of interior spaces and discuss the conclusions in the context of existing interior-design guidelines.

## Introduction

In architecture and interior design, color is an important means to modify the visual experience of interiors such that it meets the users' requirements. Apart from the aesthetic impression they create (e.g., [1–3]), surface colors of interior space affect performance (e.g., [4–8], but see [9]) as well as emotion and well-being (e.g., [10–12]). Surface color is even supposed to have the power to change the room's perceived layout. Experts in architecture and interior design propose consistent and systematic relations between the brightness (perceived luminance [13]; e.g., bright vs. dark) of a surface paint and the perceived position of this surface in space relative to the observer (e.g., [14–18]). Because home improvement and home staging have become very popular during the last decades, it is hardly surprising that expert guidelines regarding the effects of interior color on the perceived layout of interior spaces enjoy great popularity. For example, it is a common DIY recommendation to paint the rear wall of a long and narrow hallway in a darker color than the other room surfaces in order to diminish the perceived depth and, thus, optimize the perceived layout of the unfavorably shaped room (e.g., [19,20]).

To put it more precisely, architects assume that the luminance of the *bounding surfaces* (i.e., the room surface(s) that constitute the respective judged spatial dimension: the rear wall in the case of perceived depth, the side walls in the case of perceived width, or the ceiling in the case of perceived height) as well as the luminance of the *surrounding surfaces* (i.e., the room surfaces neighboring the bounding surfaces; e.g., the rear wall and the ceiling in the case of perceived width) are of importance when one wishes to adjust the perceived layout of a given room. With regard to the *bounding surfaces*, it is supposed that bright paint makes surfaces appear farther away (as compared to dark paint) and thus increases the perceived extent of the respective room dimension. A rear wall with a bright paint, for example, should then appear farther away than the same rear wall painted in a dark color. In the same line of argumentation, architects also suppose the perceived width of a given room to increase with side-wall luminance and the perceived height to increase with ceiling luminance (e.g., [14], pp. 98–100; [17], pp. 106–108; [15], p. 53). With regard to the paint of the *surrounding surfaces*, architects implicate an effect of the luminance contrast with the bounding surfaces. For the example of the narrow hallway, the shortening effect of a dark-painted rear-wall on perceived depth is supposed to be more pronounced when the side walls and the ceiling are painted in a brighter color than the rear wall (e.g., [14], pp. 98–100; [16], pp. 65–69; [15], p. 53). By the same token, when the design aim is to visually reduce the width of the room, architectural guidelines suggest to paint the side walls in a darker shade than the rear wall and the ceiling (e.g., [14], p. 100). Similar considerations apply to the optimal combination of ceiling color and wall color in order to maximize the perceived height. Here, it is suggested to combine a bright ceiling color with a slightly darker wall color in order to maximize the room's perceived height (e.g., [15], p. 53).

Taken together, experts in the field of architecture suggest that both the luminance of the bounding surfaces and the luminance of the surrounding surfaces influence the perceived extent of a given spatial dimension. Technically speaking, the latter should be an increasing function of bounding-surface luminance. The strength of this relation is assumed to be moderated by the luminance contrast between the bounding and surrounding surfaces, in the sense that a higher contrast increases the effect of bounding-surface luminance.

Is there empirical evidence for the interior-design recommendations outlined above? A series of previous studies from our lab [21–24] demonstrated that bright ceilings indeed appear higher than dark ceilings. For example, Oberfeld et al. [21] presented full-scale stereoscopic room simulations and varied the luminance of the ceiling, the walls, and the floor as well as the simulated height (290 to 310 cm) of the rooms. Participants judged the height of the rooms in units of centimeters. In line with the architects' opinion, they reported that light-gray ceilings appeared higher than medium-gray and dark-gray ceilings. However, the observed effects of wall-luminance variation did not match the predictions derived from the interior-design guidelines. The strength of the effect of ceiling luminance was not moderated by the luminance contrast between the ceiling and the walls. Instead, the data showed a purely additive effect of wall luminance: participants judged the combination of the brightest ceiling color with the brightest wall color even higher than the combination of the brightest ceiling color with any darker wall color. Moreover, the height estimates remained largely unaffected by the variation of floor luminance.

Effects of surface luminance on the perceived layout of interior spaces have also been investigated in terms of more holistic outcome measures such as spaciousness. Spaciousness refers to the perception of the room layout in terms of narrow/cramped and wide/spacious (for a more detailed discussion see, e.g., [25–27]). It has been shown that brighter surfaces, whether it be through an increase in illumination or reflectance, make rooms appear more spacious as compared to darker surfaces [2,28–30]. Thus, both the perceived extent of single room dimensions and spaciousness increase when surface luminance increases.

These results raise the question of whether the observed effect of ceiling luminance on height estimates generalizes to effects of side-wall and rear-wall luminance on the perceived width and depth of interior spaces. To answer this question, in the present study we varied the luminance of the rear wall, side-walls, and ceiling independently of each other and asked our participants to estimate the width and depth of stereoscopically presented full-scale interior spaces.

## Method

### Ethics statement

This study has been conducted in accordance with the Declaration of Helsinki and has been approved by the Institutional Review Board of the Department of Psychology at the Johannes Gutenberg-Universität Mainz (approval number 2016-JGU-psychEK-012).

### Participants

20 observers (11 women and 9 men), aged from 19 to 38 (*M* = 25.10 years, *SD* = 5.15 years) participated voluntarily in the experiment. One further participant failed to complete the experiment and was therefore excluded from all analyses. All participants gave their written informed consent. They were uninformed about the objective of the experiment. Before the experiment, potential risks were explained to the participants. After the experiment, participants were informed about the intention of the experiment.

All participants were familiar with the metric system and had normal or corrected-to-normal vision with the aid of contact lenses. Eyeglass wearers were excluded from participation due to space limitations within the head-mounted display (HMD). The visual acuity of all participants was 1.00 (Snellen fraction 6/6) or better, as confirmed by the Freiburg Visual Acuity Test (FrACT; [31]). Stereoscopic acuity was tested using a digital version of the Titmus test [32] with stereoscopic disparities of 800, 400, 200, 140, 100, 80, 60, 50, and 40 seconds of arc. In the Titmus test, at least six of the nine trials had to be answered correctly in order to qualify for participation.

### Stimuli and apparatus

**Fig 1:**
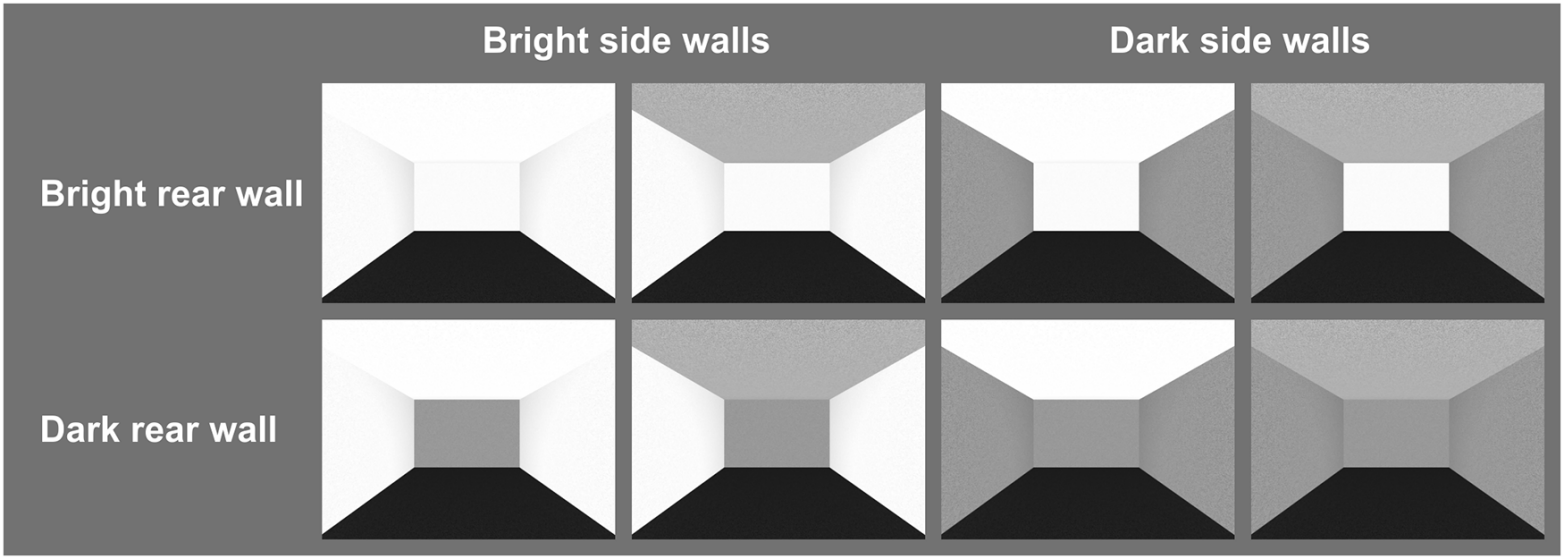
Screenshots of the display presented to the left eye of the 8 presented surface-luminance combinations. Row 1 shows rooms with bright rear walls, row 2 shows rooms with dark rear walls. Columns 1 and 2 show rooms with bright side walls; columns 3 and 4 show rooms with dark side walls. Columns 1 and 3 show rooms with bright ceilings; columns 2 and 4 show rooms with dark ceilings. In all screenshots, the simulated width, depth, and height were 4.50 m, 6.00 m, and 2.90 m, respectively.

On each trial, we presented one rectangular room stereoscopically on an HMD. Between trials, we varied the surface luminance (bright, dark) of rear wall, side walls, and ceiling independently of each other (see Fig 1), resulting in 8 different surface-luminance configurations. The colorimetric values of the presented surface colors are shown in Table 1. The colorimetric values of the dark-gray floor remained constant. All surfaces were overlaid with a fine-grained texture. The rooms were illuminated by means of a D65 [33] invisible light source positioned in the center of the room.

**Table 1:** Colorimetric values of the presented surfaces. Columns *x*, *y*, and *Y* display the CIE *xyY* values according to the 10° CIE 1964 standard observer [34], specified relative to a D65 white point [33].

Surface	Luminance	*x*	*y*	*Y* (cd/m^2^)
**Ceiling**	Low	0.30	0.33	34.87
	High	0.30	0.34	82.64
**Rear wall**	Low	0.30	0.33	25.46
	High	0.30	0.34	82.08
**Side walls**	Low	0.30	0.33	25.45
	High	0.30	0.34	82.11
**Floor**	-	0.28	0.32	0.83

**Fig 2:**
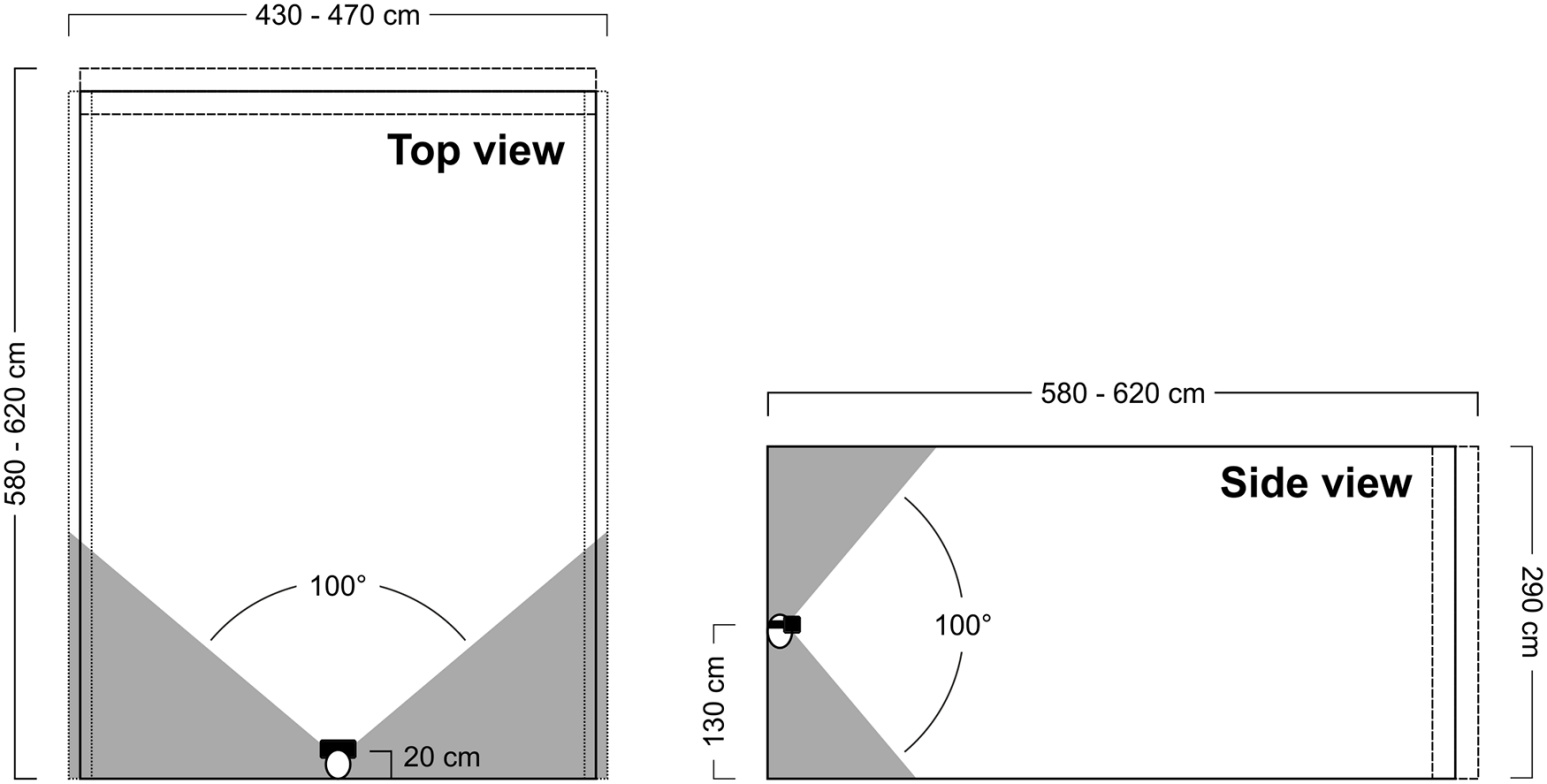
Top view (left-hand side) and side view (right-hand side) of the observer's virtual position relative to the simulated rooms. Within the simulated rooms, the gray-shaded areas were not visible to the observer.

Depending on the experimental block, we additionally varied the physical width (4.30, 4.50, and 4.70 m) or depth (5.80, 6.00, and 6.20 m) of the simulated rooms. In trials where the width was varied (width blocks), the depth was set constant at the medium value of 6.00 m. When depth was varied (depth blocks), the width was set constant at the medium value of 4.50 m. In all trials, the ceiling height was set constant at 2.90 m (see Fig 2, top and side view).

The observer’s virtual position remained constant at 20 cm in front of the simulated room’s invisible front wall, horizontally centered between the left and the right side wall (see Fig 2, top view). The virtual viewing direction was horizontally and vertically perpendicular to the room’s rear wall. Subjects were instructed that their virtual position was like sitting on a chair and leaning with their back against the horizontal center of the simulated room’s front wall.

The geometric field of view (gFOV; enclosed visual angle of the projection) was approximately 100° horizontally × 100° vertically. The virtual field of view (vFOV; visible area of the simulated room) corresponded to the gFOV (see Fig 2, top and side view). Subjects’ head position was fixed by means of a chin rest. The virtual eye height was set constant at 1.30 m (see Fig 2, side view) and corresponded to physical eye height. We did not use head-tracking.

The stimuli were generated using Vizard 5 [35] on a Core i5 computer with an NVIDIA QuadroFX1800 graphics board and presented on an Oculus Rift DK2^™^ (second developer's kit). The HMD had a resolution of 960 × 1,080 pixels per eye (horizontal × vertical), a color resolution of 8 bits per channel, and a refresh rate of 75 Hz. The individual inter-pupillary distance of each subject was measured with the aid of a pupil distance meter (bon PD-2) before the experiment and taken into account when computing the binocular disparity of the images presented to the left and right eye.

### Design and procedure

Subjects judged the width of the simulated rooms in one half of the blocks (width blocks) and the depth in the other half (depth blocks). On each trial, the simulated room was presented for 5 seconds. Subsequently, subjects provided a verbal estimate of the width / depth of the presented room in units of meters and centimeters. No time limit was given for the response. The experimenter entered the estimate using the computer keyboard and then advanced to the next trial.

Width and depth blocks were presented in alternating order. The initial block type (width vs. depth) was balanced between subjects. In each block, we presented the 8 different surface-luminance configurations combined with the 3 different spatial extents of width (width blocks) or depth (depth blocks) in a factorial design, resulting in 24 trials per block. The experiment consisted of 2 sessions of 10 blocks each, such that each condition (judged dimension × surface-luminance configuration × spatial extent) was presented 10 times.

In session 1, prior to the first width and depth block, subjects completed each six training trials (drawn at random from the 24 trials). The training trials were not taken into account in the data analyses. The time interval between sessions 1 and 2 was minimally 1 hour and maximally 1 week. In total, the experiment consisted of 492 trials and lasted approximately 130 min (70 min for session 1 and 60 min for session 2).

Subjects were tested individually in a dimly lit rectangular office room with approximately 12.50 m^2^ surface area and 2.90 m ceiling height. Due to the use of the HMD, the office room was not visible to the participants during the trials.

## Results

For both dependent measures, we analyzed the subjects’ mean estimates in the 24 experimental conditions, averaged across the 10 repetitions per condition. Data were corrected in two steps prior to the analyses. First, 1 width estimate and 6 depth estimates were excluded because the experimenter had made an error when entering the participant’s estimate. Second, individual means were corrected for outliers using the Tukey criterion. Estimates more than 1.5 times the interquartile range below the first quartile or above the third quartile were classified as outliers and excluded from further analyses. This affected only 101 of the remaining 4799 width estimates (2.1%) and 136 of the remaining 4794 depth estimates (2.8%). We calculated one repeated measures analysis of variance (rmANOVA) for the width estimates and another for the depth estimates, using a univariate approach with Huynh and Feldt [36] correction for the degrees of freedom. The correction factor $\tilde{\varepsilon }$ is reported. In the analysis of the width estimates, the within-subjects factors were side-wall luminance, rear-wall luminance, ceiling luminance, and physical width. In the analysis of the depth estimates, the within-subjects factors were side-wall luminance, rear-wall luminance, ceiling luminance, and physical depth. The α-level was .05 in all analyses.

### Width estimates

**Fig 3:**
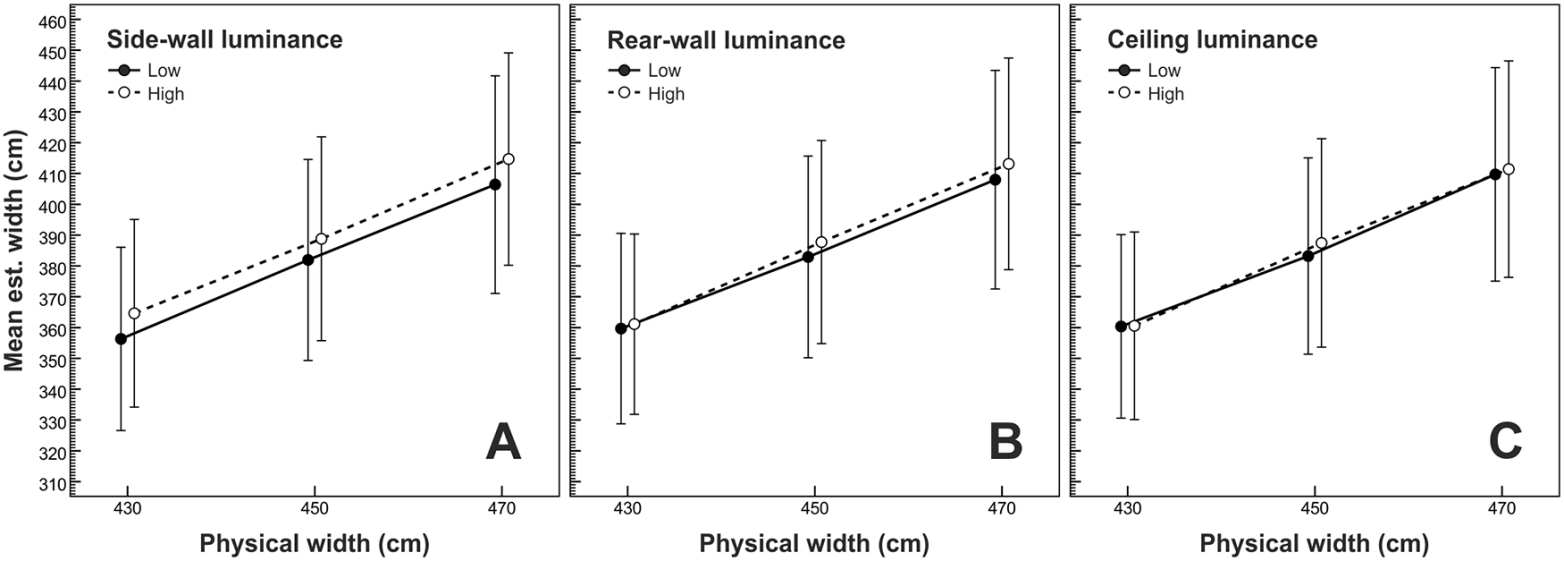
Mean width estimates as a function of physical width. The data lines in panels A, B, and C show the effects of side-wall luminance, rear-wall luminance, and ceiling luminance, respectively. Error bars show ± 1 standard error of the mean (*SEM*) of the 20 subjects in each condition.

The mean width estimates are depicted in Fig 3. The rmANOVA showed a significant effect of side-wall luminance, *F*(1,19) = 17.85, *p* < .001, η^2^_p_ = .48, Cohen's [37] *d*_*z*_ = 0.98. On average, rooms with bright side walls appeared 7.88 cm wider than rooms with dark side walls (see Fig 3, panel A). In contrast, the mean width estimates remained largely unaffected by the variation of rear-wall luminance (see Fig 3, panel B), *F*(1,19) = 2.38, *p* = .140, η^2^_p_ = .11, *d*_*z*_ = 0.38, and ceiling luminance (see Fig 3, panel C), *F*(1,19) = 1.30, *p* = .268, η^2^_p_ = .06, *d*_*z*_ = 0.25. In addition, the rmANOVA showed a significant effect of physical width, *F*(2,38) = 30.33, *p* < .001, η^2^_p_ = .61, $\tilde{\varepsilon }=.53$. The width estimates increased as physical width increased (see Fig 3), indicating that subjects really judged the width of the presented rooms. The width estimates under the different levels of simulated width differed significantly from each other, as witnessed by post-hoc paired-samples *t*-tests (two-tailed) with Hochberg [38] correction for multiple testing: $\overset{-}{\Delta }(mean\;difference)=24.87\;cm$, *d*_*z*_ = 1.13 for the comparison 450 cm vs. 430 cm; $\overset{-}{\Delta }=25.18\;cm$, *d*_*z*_ = 1.31 for the comparison 470 cm vs. 450 cm.

**Fig 4:**
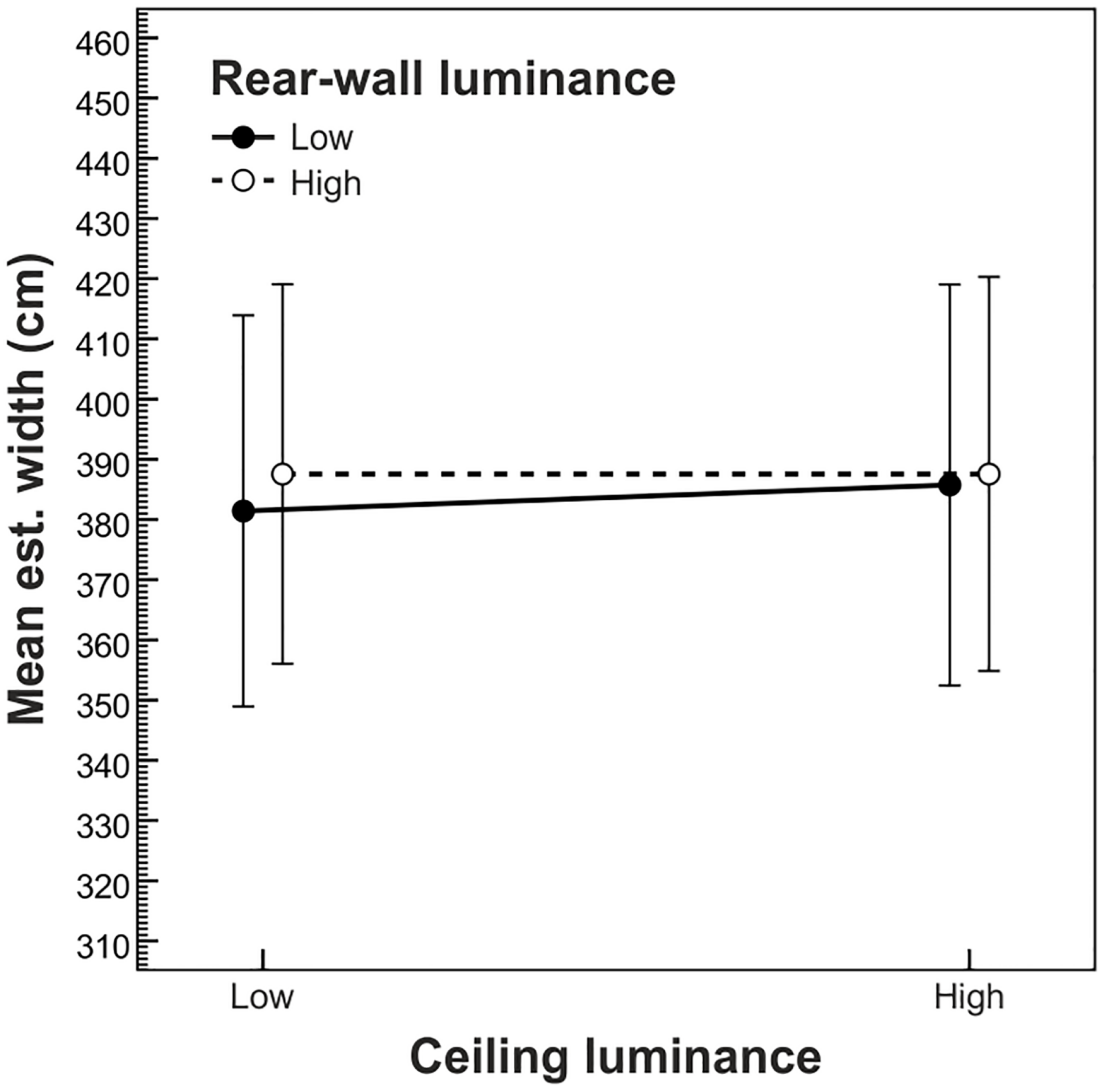
Mean width estimates as a function of ceiling luminance and rear-wall luminance. Error bars show ± 1 *SEM* of the 20 subjects in each condition.

The rear-wall luminance × ceiling luminance interaction reached significance, *F*(1,19) = 5.70, *p* = .027, η^2^_p_ = .23. As can be seen in Fig 4, we observed slightly smaller width estimates when both the rear wall and the ceiling were dark as compared to all other combinations of rear-wall luminance and ceiling luminance. All other effects were not significant (all *p*-values ≥ .166).

Across all experimental conditions, the mean perceived width was 385.38 cm (*SD* = 144.49 cm). Relative to the mean physical width of 450 cm, this indicates an underestimation of 14.36% on average.

### Depth estimates

**Fig 5:**
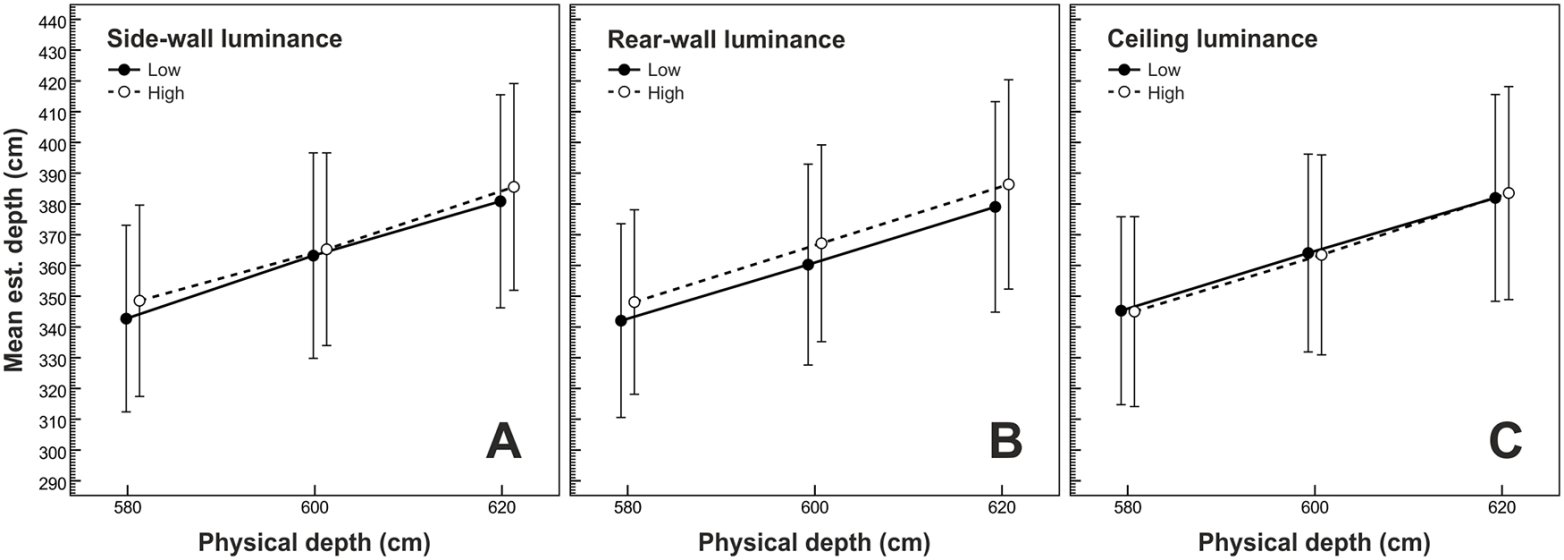
Mean depth estimates as a function of physical depth. The data lines in panels A, B, and C show the effects of side-wall luminance, rear-wall luminance, and ceiling luminance, respectively. Error bars show ± 1 *SEM* of the 20 subjects in each condition.

The mean depth estimates are depicted in Fig 5. The rmANOVA showed a significant effect of rear-wall luminance, *F*(1,19) = 7.52, *p* = .013, η^2^_p_ = .28, *d*_*z*_ = 0.62. Perceived depth increased with increasing rear-wall luminance (see Fig 5, panel B). On average, bright rear walls appeared 6.84 cm farther away than dark rear walls. In contrast, the depth estimates remained largely unaffected by the variation of side-wall luminance (see Fig 5, panel A), *F*(1,19) = 2.41, *p* = .137, η^2^_p_ = .11, *d*_*z*_ = 0.32, and ceiling luminance (see Fig 5, panel C), *F*(1,19) = 0.01, *p* = .919, η^2^_p_ < .01, *d*_*z*_ = 0.02. The effect of physical depth was significant, *F*(2,38) = 27.59, *p* < .001, η^2^_p_ = .59, $\tilde{\varepsilon }=.52$. Comparable to the results for the width estimates, perceived depth increased as physical depth increased (see Fig 5). The post-hoc paired-samples *t*-tests showed that the depth estimates under the different levels of simulated depth differed significantly from each other: $\overset{-}{\Delta }=18.61\;cm$, *d*_*z*_ = 1.18 for the comparison 600 cm vs. 580 cm; $\overset{-}{\Delta }=18.99\;cm$, *d*_*z*_ = 1.14 for the comparison 620 cm vs. 600 cm.

**Fig 6:**
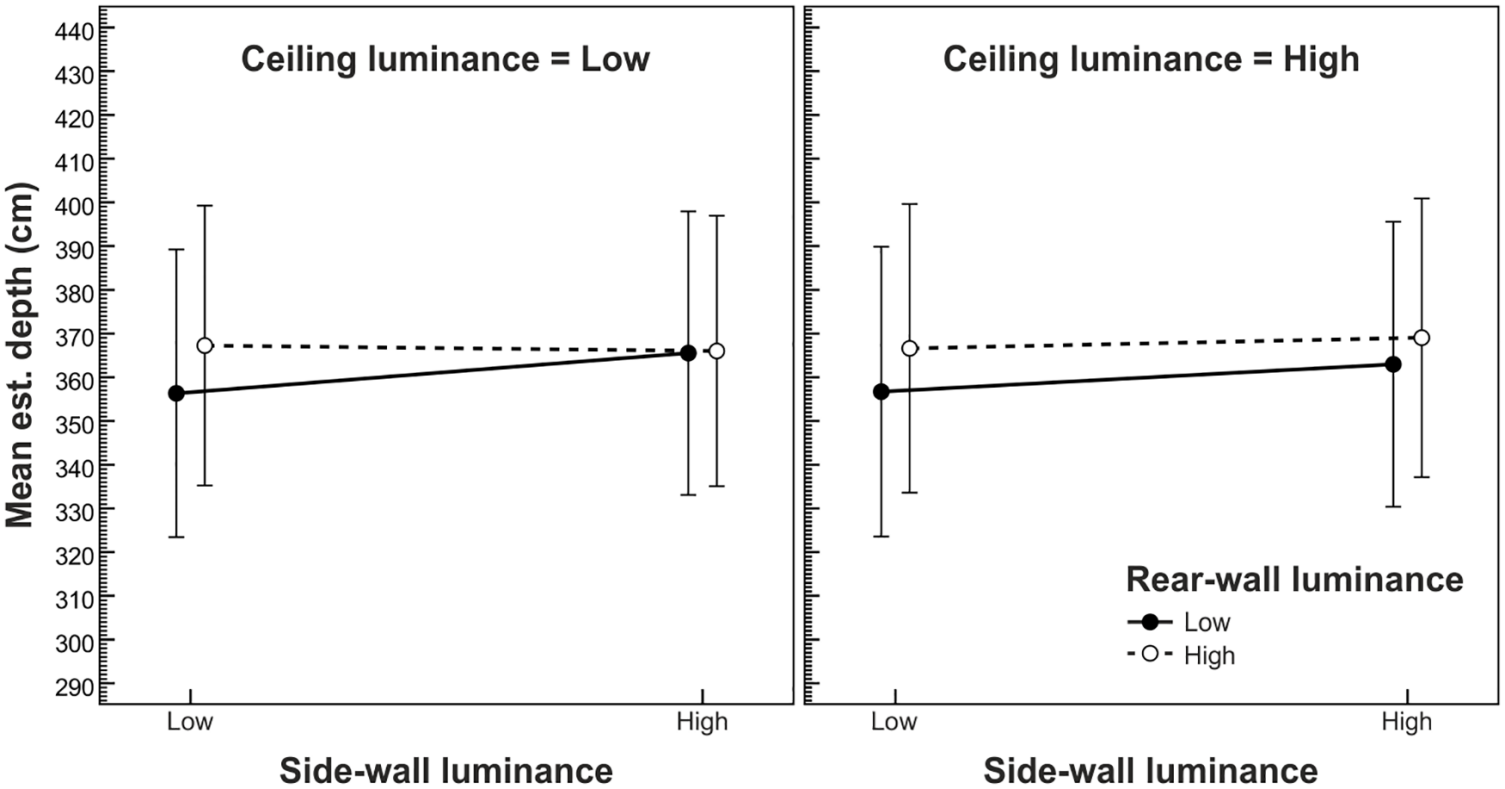
Mean depth estimates as a function of side-wall luminance, ceiling luminance, and rear-wall luminance. Error bars show ± 1 *SEM* of the 20 subjects in each condition.

In the rmANOVA, the rear-wall luminance × side-wall luminance interaction and the rear-wall luminance × side-wall luminance × ceiling luminance interaction were also significant, *F*(1,19) = 4.99, *p* = .038, η^2^_p_ = .21 and *F*(1,19) = 4.52, *p* = .047, η^2^_p_ = .19, respectively. As shown in Fig 6, the depth estimates were more strongly influenced by the variation of rear-wall luminance when the side walls were dark as compared to bright. In addition, this interaction was more pronounced when the ceiling was dark (see Fig 6, left panel) as compared to bright (see Fig 6, right panel). All other effects were not significant (all *p*-values ≥ .246).

Across all experimental conditions, the mean perceived depth was 363.74 cm (*SD* = 142.87 cm), indicating that participants underestimated the mean physical depth (600 cm) by 39.38% on average. Comparing our results for the width and depth estimates, participants showed a more pronounced underestimation of depth than of width (the average width underestimation was 14.36%). In addition, the observed underestimation of width and depth exceeds the amount of underestimation reported in previous studies concerned with the perceived height of interior spaces [21–24]. Note that this is in line with the results from von Castell et al. [25] whose subjects estimated the width, depth, and height of square stereoscopic room simulations. They reported the most pronounced underestimation for depth, followed by width, and height. We will return to this topic in the Discussion section.

## Discussion

In the present study, we varied the luminance of interior-space surfaces (i.e., ceiling, side walls, and rear wall) independently of each other. Participants' estimates of the width and depth of the presented rooms indicated that a bright paint of the *bounding surfaces*, which constitute the respective judged spatial dimension (e.g., the side walls in the case of judged width), enlarges the perceived extent of this dimension: a bright rear wall makes a room appear deeper and bright side walls make a room appear wider as compared to a dark surface paint. Cohen's [37] *d*_*z*_ indicated a medium (depth, *d*_*z*_ = 0.62) to strong (width, *d*_*z*_ = 0.98) effect size. It is interesting to compare these effect sizes with the effect sizes of the variation in simulated width and depth. Be reminded that we had varied the simulated width and depth in three steps of 20 cm around 450 cm and 600 cm, respectively. On average, we found a *d*_*z*_ of 1.22 ([1.31 + 1.13] / 2) for the effect of a 20 cm increase in simulated width on the width estimates, and a *d*_*z*_ of 1.16 ([1.18+ 1.14] / 2) for the effect of a 20 cm increase in simulated depth on the depth estimates. Thus, an increase in side-wall luminance yielded 80% (0.98 / 1.22 · 100%) of the effect size of a 20 cm increase of simulated width on the width estimates. An increase in rear-wall luminance yielded 53% (0.62 / 1.16 · 100%) of the effect size of a 20 cm increase of simulated depth on the depth estimates. Put differently, an increase in side-wall luminance yielded the same effect on the width estimates as a 16.0 cm (0.80 · 20 cm) increase in simulated width, and an increase in rear-wall luminance yielded the same effect on the depth estimates as a 10.6 cm (0.53 · 20 cm) increase in simulated depth. The luminance variation of the *surrounding surfaces* yielded merely subordinate effects on both the width and depth estimates. We found smaller width estimates when both the ceiling and the rear wall were dark as compared to all other combinations of rear-wall and ceiling luminance. In addition, bright side walls reduced the perceived decrease of room depth produced by a dark rear wall, especially when the ceiling was dark. All other effects regarding the luminance of the surrounding surfaces did not reach significance. In sum, the luminance of the surrounding surfaces had either a small additive effect, as in the case of perceived width, or a small moderating effect, as in the case of perceived depth. Note, however, that the luminance effect of the bounding surfaces remained consistent across all experimental manipulations: brighter bounding surfaces appeared to be farther away or farther apart.

How do the results of the present study integrate into previous findings? Table 2 provides a short overview of the results from the present study and previous studies concerned with luminance effects on the perceived extent of spatial dimensions. We first consider effects of *bounding-surface luminance* (highlighted by a gray background in Table 2). Across all studies and spatial dimensions, the data show an increase in perceived spatial extent (cf. column ${\overset{-}{\Delta }}_{bd}$) with increasing bounding-surface luminance. With one exception (achromatic condition in [24]), the gain in perceived extent reached significance with medium to large effect sizes. Second, the variation of the luminance of the *surrounding surfaces* did only influence the perceived extent of the height dimension: Oberfeld et al. [21] and Oberfeld and Hecht [22] reported an approximately additive effect of wall-luminance on the perceived height of interior spaces. Third, and not depicted in Table 2, the effect of bounding-surface luminance was merely slightly influenced by the luminance contrast with the surrounding surfaces. In the present study, we found only side-wall luminance to moderate the effect of rear-wall luminance on perceived depth, especially when the ceiling was dark (see above). In addition, Oberfeld et al. [21] and Oberfeld and Hecht [22] reported the effect of ceiling luminance on the perceived height of interior spaces to be virtually unaffected by wall luminance. Thus, considering the results from Table 2, the following picture emerges: a bright room surface appears more distant to the observer as compared to a dark room surface, mostly independent of its orientation in space and the luminance contrast between it and its surrounding surfaces.

**Table 2:** Overview of effects of surface luminance on the perceived layout of interior space, as a function of the room surface that was varied in luminance, and the room dimension that was judged by the observers. $\bar{S}$ indicates the mean simulated size of the judged spatial dimension. *Y*_*b*_ and *Y*_*d*_ indicate the luminance of the brightest and the darkest presented surface color, respectively. The difference in perceived brightness (*ΔB*), the mean difference in perceived spatial extent (${\overset{-}{\Delta }}_{b-d}$), and Cohen's [37] *d*_*z*_ are reported for the comparison of the brightest and darkest presented surface color. We calculated the difference in perceived brightness between the dark and the bright stimulus by ΔB = *Y*_*b*_^0.33^−*Y*_*d*_^0.33^, where the power function links the perceived brightness *B* to the physical luminance *Y* (e.g., [39], see also [40,41]). Bold *d*_*z*_-values indicate significant effects (*p* < .05). Effects of bounding-surface luminance are highlighted by a gray background.

Surface with luminance variation	Judged dimension	Study	Experiment	$\bar{\text{S}}$ (cm)	*Y*_*b*_ (cd m^-2^)	*Y*_*d*_ (cd m^-2^)	*ΔB*	${\overset{-}{\Delta }}_{\text{b}-\text{d}}$ (cm)	*d*_*z*_
Ceiling	Height	Oberfeld et al. (2010) [21]	1	300	^a^	^a^		8.56	**0.66**
			2	300	^a^	^a^		3.64	**0.50**
		Oberfeld & Hecht (2011) [22]	2	300	^a^	^a^		3.76	**0.63**
			3	300	^a^	^a^		7.63	**0.46**
		von Castell et al. (2017) [23]	3 (matching task ^b^)	300	17.51	1.05	1.56	1.93	**0.79**
			3 (verbal estimates)	300	17.51	1.05	1.56	3.38	**0.51**
		von Castell et al. (in press) [24]	Chromatic colors	300	13.74	4.76	0.70	1.40	**0.47**
			Achromatic colors	300	13.78	4.70	0.70	1.17	0.28
	Width	Oberfeld & Hecht (2011) [22]	3	450	^a^	^a^		−6.07	0.38
		Present study		450			1.06	2.14	0.25
	Depth	Present study		600	82.64	34.87	1.06	0.14	0.02
Side walls	Height	Oberfeld et al. (2010) [21] ^c^		300	^a^	^a^		9.45	**0.62**
		Oberfeld & Hecht (2011) [22] ^c^	2	300	^a^	^a^		8.69	**0.77**
	Width	Present study		450	82.11	25.45	1.37	7.88	**0.98**
	Depth	Present study		600	82.11	25.45	1.37	4.09	0.32
Rear wall	Height	Oberfeld et al. (2010) [21] ^c^		300	^a^	^a^		9.45	**0.62**
		Oberfeld & Hecht (2011) [22] ^c^	2	300	^a^	^a^		8.69	**0.77**
	Width	Present study		450	82.08	25.46	1.37	3.97	0.38
	Depth	Present study		600	82.08	25.46	1.37	6.84	**0.62**

Note:

^a^ No luminance measurement available.

^b^ Results for the comparison stimulus (pillar) presented at a distance of 4.50 m from the observer (for a discussion see [23]).

^c^ No independent variation of side-wall and rear-wall luminance.

In the Introduction, we had derived two rules from the architectural experts' guidelines: First, the perceived extent of a given spatial dimension is a positive function of bounding-surface luminance. Second, the strength of this relation is moderated by the luminance contrast between the bounding and the surrounding surfaces in the sense that a higher contrast increases the effect of variations in bounding-surface luminance. How do our results fit with these predictions? Our results do clearly support the first prediction: brighter surfaces appeared farther away than darker surfaces. However, our results do not support the second prediction. We found the effect of bounding-surface luminance to be mostly independent of the luminance of the surrounding surfaces, notwithstanding our finding that bright side walls mitigated the reduction in perceived depth due to a dark rear-wall paint, especially when the ceiling was dark. Note that the direction of this interaction is not compatible with the second design guideline: if one follows the suggestion that an increase in luminance contrast between bounding surface and surrounding surfaces increases the effect of bounding surface luminance, bright side-walls should have reinforced the reduction in perceived depth due to a dark rear wall paint. In sum, our findings only partly support the guidelines suggested by experts in the field.

The discrepancy between empirical data and predominant opinion in the application area raises the question about the applicability of our findings to the real world. All results in Table 2 are from experiments presenting stereoscopic simulations of interior spaces. Despite many advantages, such as an almost perfect stimulus control and a highly economic generation of the rather complex stimulus material, it is obvious that simulations have limitations in comparison to real-world scenes. Most prominently, to control for possible interactions with further room properties such as window arrangement, daylight condition, furniture arrangement, and so on, we presented rather minimalistic simulations of unfurnished and windowless interior spaces, which questions the full ecological validity of the stimuli. However, we argue that the control of potential confounding variables outweighs potential limitations regarding the ecological validity of the stimulus material. In addition, even though rather clear-cut in structure, the simulations unambiguously evoked the perception of an interior space. As a second point, it should be noted that stereoscopic simulations provide less accurate depth cues as compared to real scenes (cf. [42]). Many studies regarding the perception of visual extent in virtual environments have reported a pronounced underestimation of stereoscopically presented spatial extent as compared to estimates of spatial extent in real environments, in particular, but not exclusively [25,43], for estimated depth (e.g., [43–50]; for a review see [51]). Such pictorial displays do suffer from sagittal foreshortening, in particular if the pictorial space is empty (see, e.g., [52,53]) and no feedback is provided (e.g., [54,55]; but see [56]). In line with these findings, we also observed a consistent underestimation of the presented spatial extent for both the width (cf. [25,43]) and the depth estimates in the present study. However, this does not have a systematic bearing on the effects reported here.

Due to the within-subjects design we used in the current experiment, all reported effects refer to deviations from the observers' individual baseline due to the variation of the independent variables. For example, our finding that brighter surfaces appear farther away than darker surfaces is based on the perceived extent of room dimensions bordered by bright surfaces *relative* to the perceived extent of equally sized room dimensions bordered by dark surfaces. Thus, the direction and slope of the reported effects remained unaffected by the *absolute* level of estimates. Following this consideration, we argue that the overall underestimation of spatial extent in virtual environments is not at odds with the transferability of our results to real-world environments. Having said this, the replication of our findings in real-world scenes and under more ecological conditions (windows, furnishing, etc.) would be desirable.

A further limitation concerns potential effects of the remaining color dimensions, which can be described in terms of hue (e.g., red, green, blue) and saturation (i.e., the distance to an achromatic stimulus of the same brightness, i.e., a neutral gray) (cf. [57–59]). We only used achromatic surface colors (shades of gray) in the present study. Thus, we cannot evaluate potential effects of chromatic wall and ceiling colors on the perceived width and depth of interior spaces. With regard to perceived height, however, von Castell et al. [24] varied the luminance (bright, dark), hue (red, green, blue), and saturation (achromatic, low, high) of the ceiling paint independently of each other and could replicate the luminance effect obtained for achromatic ceiling colors. In addition, they only found a small effect of hue (in the sense that green ceilings appeared somewhat lower as compared to all other chromatic and achromatic ceilings) and virtually no effect of saturation on the height estimates, which indicates that effects of ceiling color on the perceived height of interior spaces are mainly driven by the luminance dimension of color. We expect the effects of surface color on the perceived width and depth to be also mainly driven by differences in the luminance of the surface paint. If this is the case, then the chromatic characteristics of the room paint can be freely chosen in order to maximize the usability of the room in terms of the aesthetic experience, measures of well-being (e.g., in counseling or hospital rooms), or performance outcomes (e.g., in classrooms or office rooms). However, the effects of surface hue or saturation need further investigation.

Which practical implications can be derived from our results? First, in order to make a room appear maximally large, our data plead for painting all walls and the ceiling in the brightest possible color. Second, our results also indicate that effects due to the luminance contrast between adjacent room surfaces, such as between the rear wall and side walls or between the walls and the ceiling, only play a minor part and should not be overstated.

## Supporting information

S1 File Raw data of the width and depth estimates. Archive file containing the raw datasets (i.e., uncorrected and not aggregated across repetitions per condition) for the width estimates (widthEstimates.csv) and depth estimates (depthEstimates.csv). When the variable 'typoExperimenter' has the value 'true', this indicates that the experimenter noted an uncorrected typo for the entered estimate on the given trial. These trials were excluded before performing the outlier analysis. When the variable 'outlier' has the value 'true', this indicates that the estimate on the given trial was more than 1.5 times the interquartile range below the first quartile or above the third quartile, relative to the set of ten trials collected for the given combination of subject and experimental condition (judged dimension × surface-luminance configuration × spatial extent). These trials were excluded from our data analyses.
(ZIP)
